# Portal Hypertension, Nodular Regenerative Hyperplasia of the Liver, and Obstructive Portal Venopathy due to Metastatic Breast Cancer

**DOI:** 10.1155/2013/826284

**Published:** 2013-07-31

**Authors:** Andrew T. Turk, Matthias J. Szabolcs, Jay H. Lefkowitch

**Affiliations:** Department of Pathology and Cell Biology, Columbia University, New York, NY 10032, USA

## Abstract

Nodular regenerative hyperplasia (NRH) of the liver is associated with noncirrhotic portal hypertension, rheumatologic and hematologic disorders, administration of certain drugs, and other underlying conditions. This report describes a 64-year-old man with clinically presumed cirrhosis who presented to our institution with coffee-ground emesis, esophageal varices, ascites, and encephalopathy. Eleven years earlier he had been treated for breast cancer with mastectomy and chemo-radiotherapy. He died suddenly, and the autopsy showed no evidence of cirrhosis but instead demonstrated NRH with extensive emboli of recurrent breast carcinoma within the portal vein and its intrahepatic branches. Neoplastic occlusion of the portal vein as a cause of presinusoidal noncirrhotic portal hypertension has not previously been reported for metastatic breast carcinoma. This case highlights the importance of obstructive portal venopathy in the pathogenesis of NRH as well as the diagnostic difficulties that may be encountered in determining the cause of portal hypertension.

## 1. Report of a Case

The patient was a 64-year-old man with a history of invasive carcinoma of the left breast 11 years earlier which had been treated by mastectomy (4 of 15 lymph nodes involved), radiotherapy, and chemotherapy. There was a maternal history of breast cancer. There was no known tumor recurrence during the subsequent 10 years. Nine months before his terminal admission, during evaluation for constipation, he underwent an abdominal/pelvic CT scan that showed “mild nodularity of the liver parenchyma,” raising the possibility of cirrhosis (which was also considered likely because of a history of alcohol use and risk factors for nonalcoholic fatty liver disease including hyperlipidemia and diabetes). Seven months later the patient had developed ascites and encephalopathy that resolved with rifaximin/lactulose treatment. He was hospitalized elsewhere for several days of coffee-ground emesis and then was transferred to our medical center for further care and evaluation for liver transplantation. Esophagogastroduodenoscopy demonstrated grade II esophageal varices, and banding was performed. Serum liver function tests showed a disproportionately elevated alkaline phosphatase of 405 U/L (normal < 140) and gamma glutamyl transferase (GGT) of 215 U/L (normal < 65). Total bilirubin and aspartate aminotransferase (AST) were minimally elevated at 1.5 mg/dL (normal < 1.0) and 57 U/L (normal < 40), respectively. Alanine aminotransferase (ALT), albumin, and total protein levels were normal. MRI showed portal vein thrombosis and cavernous transformation. Bone scan performed three days later showed multiple possible metastatic bony lesions. He was deemed ineligible for liver transplantation. The evening before his death he developed diarrhea and a temperature of 100.1 degrees Fahrenheit and the next morning was found unresponsive in his room. He was not resuscitated, as per his and his family's request.

## 2. Autopsy Findings

**Figure 1:**
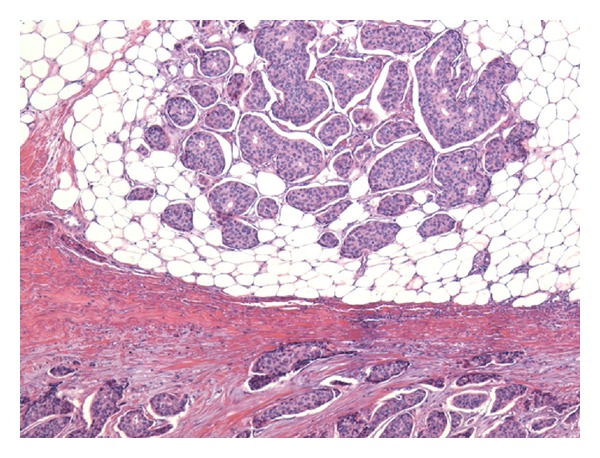
Mastectomy specimen with invasive ductal carcinoma. Duct-like structures and nests are seen invading the breast stroma and fat. (Hematoxylin and eosin stain, original magnification ×100.)

**Figure 2:**
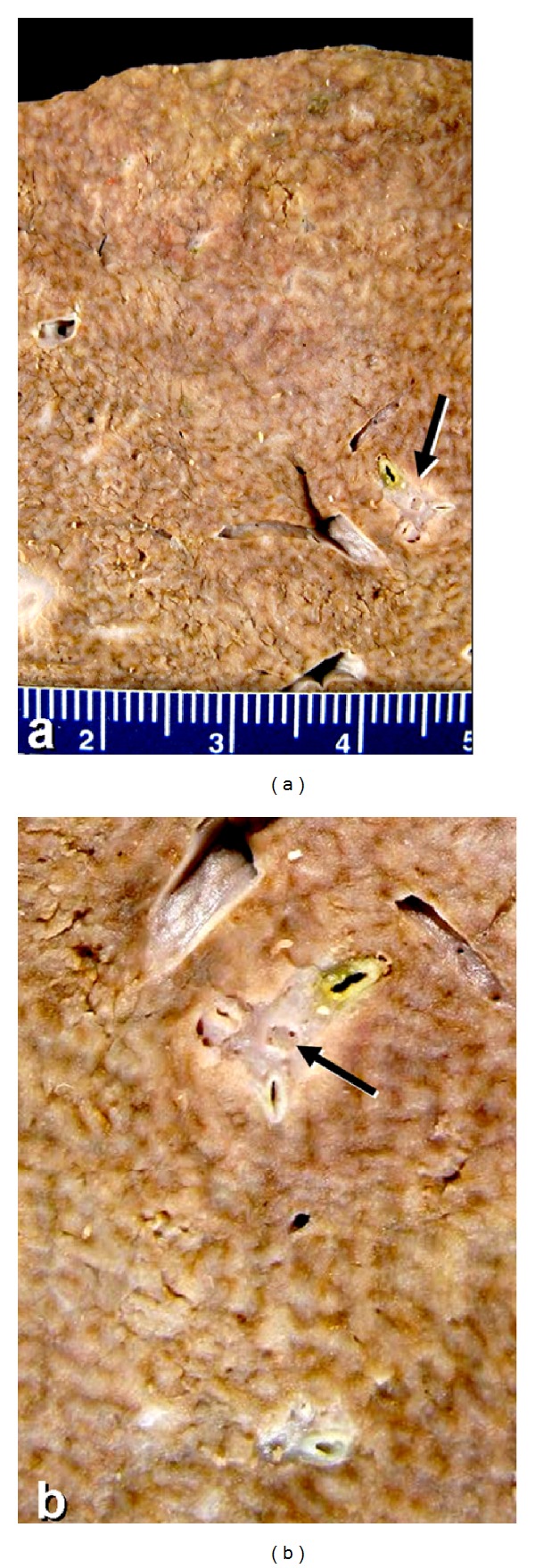
(a) Postmortem liver with nodular regenerative hyperplasia (NRH). The cut surface demonstrates small nodules <3 mm in size without surrounding fibrosis. The portal tract at the arrow is enlarged in (b). The ruler is in centimeters. (b) The small regenerative nodules of NRH are apparent. The portal tract at center shows a putty-like tumor thrombus of breast carcinoma within the portal vein branch (arrow).

**Figure 3:**
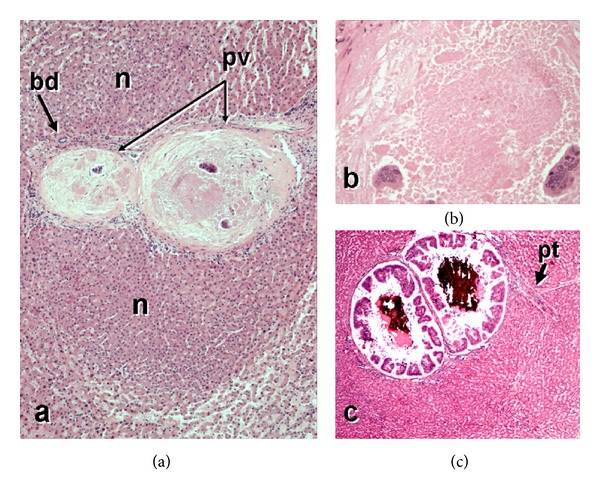
(a) Obstructive portal venopathy due to metastatic breast carcinoma is seen at low power and is associated with periportal regenerative nodules (n), without fibrosis (nodular regenerative hyperplasia). The portal tract is shown at center, including its bile duct (bd) as well as portal vein branches (pv) filled with necrotic tumor. (b) The tumor thrombus within the portal vein branch shows extensive necrosis, with only a few clusters of preserved tumor cells at the periphery. (c) This portal tract (pt) shows breast carcinoma within the portal vein branch, growing with peripherally arranged tubular-glandular units and prominent central necrosis with calcification. (Hematoxylin and eosin stain, original magnification (a) ×40; (b) ×200; (c) ×40.)

Postmortem examination demonstrated recurrent breast carcinoma with diffuse carcinomatosis involving multiple vertebrae, the right pectoralis muscle and right lung, diaphragm, and peritoneum. The tumor microscopically closely resembled the original invasive ductal carcinoma of the breast, microscopic slides of which were obtained and reviewed (Figure 1), and was consistent with breast carcinoma by immunohistochemistry. The peritoneum contained 4500 cc of ascites. The liver did not show cirrhosis but instead demonstrated the gross and microscopic features of NRH, with diffuse 1–3 mm regenerative nodules and no surrounding fibrosis (Figure 2). Close gross inspection of the major portal vein as well as of the intrahepatic portal vein branches showed putty-like tan-grey intravascular plugs of carcinoma, consistent with diffuse portal venous tumor embolization (Figure 2(b)). On microscopy, the affected portal veins were obliterated by a combination of necrotic tumor and reactive fibrosis, with only focally viable carcinoma (Figure 3). The periportal liver parenchyma near the occluded portal vein branches showed regenerative nodules composed of thickened hepatocyte plates bulging into centrilobular regions where there were patchy sinusoidal dilatation and liver-cell plate atrophy (Figure 3(a)). There was no steatosis, steatohepatitis, or centrilobular fibrosis. There was mild splenomegaly (373 g, normal = 150), and banded esophageal varices were identified. Postmortem blood culture grew *E. coli *suggesting that sepsis may have been a significant contributory factor to the patient's sudden death.

## 3. Comment

The autopsy in this case demonstrated three uncommon conditions: (1) male breast cancer; (2) portal vein tumor thrombosis due to a malignancy other than primary hepatocellular, bile duct, or pancreatic carcinoma; and (3) nodular regenerative hyperplasia of the liver. Male breast cancer is a rare disease comprising less than 1% of all breast cancer patients [1]. Male breast carcinoma shows a unimodal peak at age 71 and is histologically most often invasive ductal carcinoma [1, 2], as in this case. Prognosis is negatively influenced by tumor size, lymph node involvement, and increasing stage [1]. In this patient, 4 of 15 axillary lymph nodes were positive for tumor at the time of mastectomy 11 years earlier and, despite chemo- and radiotherapy and a lengthy period of quiescence, the tumor recurred with extensive portal venous embolization and associated NRH.

In contrast to these unusual postmortem findings, the majority of this patient's presenting signs and symptoms during the year prior to his death were those of the considerably more common clinical problem of portal hypertension (including ascites, encephalopathy, and esophageal varices). However, the portal hypertension in this case was not due to cirrhosis (which is the major cause of portal hypertension in Western countries), but was due to noncirrhotic portal hypertension associated with NRH and portal vein tumor thrombi. Other forms of liver pathology associated with non-cirrhotic portal hypertension include schistosomiasis (“clay pipestem fibrosis”), idiopathic portal fibrosis, hepatoportal sclerosis, and partial nodular transformation [3]. Although this man had been clinically presumed to have cirrhosis based on a history of alcohol use combined with risk factors for nonalcoholic fatty liver disease (including hyperlipidemia and diabetes), autopsy demonstrated a form of presinusoidal noncirrhotic portal hypertension due to extensive portal vein obstruction by emboli of recurrent breast carcinoma. Portal vein tumor thrombosis due to carcinoma is most often in the setting of gastrointestinal tract tumors, primarily hepatocellular, bile duct, or pancreatic carcinoma [4, 5]. However, other primary malignancies have been associated with portal venous metastasis and intraluminal tumor thrombosis including lung adenocarcinoma, renal cell carcinoma, melanoma, and lymphoma [6–10]. To our knowledge, portal vein intraluminal tumor thrombosis due to carcinoma of the breast has not previously been reported. While breast carcinoma often metastasizes to the liver, when portal vein obstruction comes to clinical attention and is confirmed radiologically, the portal vein obstruction is most often because of *extravascular* compression and narrowing of the vein and its branches by liver parenchymal metastases, rather than by actual intraluminal tumor thrombi as seen in the present case [7].

The pathogenesis of NRH is related to altered blood flow within the liver and has been studied extensively by Wanless and colleagues [3, 11, 12]. The presence of portal vein obstruction by thrombus (or tumor thrombus, as in this report) has been stressed, with the resultant obstructive portal venopathy producing regions of decreased parenchymal perfusion and associated atrophy. Such regions alternate with areas of normal or increased perfusion, thereby resulting in compensatory hyperplasia in the form of periportal regenerative nodules. From the clinical vantage point, this patient's disproportionate elevation of serum alkaline phosphatase (AP) (and gamma glutamyl transferase) is consonant with the reported 25% of patients with NRH who show such AP elevations [12]. The fourfold AP elevation, in the absence of known biliary tract obstructive disease, would be an unusual finding in individuals with more prevalent types of cirrhosis, such as those due to chronic hepatitis or alcoholic and nonalcoholic fatty liver disease. The possibility of NRH might have been raised based on that finding, as well as on the preservation of hepatic synthetic function in this patient (with normal serum albumin and total protein levels). In summary, this case represents the confluence of several uncommon conditions (recurrence of breast cancer in a man over a decade after his mastectomy, widespread portal venous tumor thrombi with obstructive venopathy, and nodular regenerative hyperplasia of the liver as a consequence of the portal vein obstructive lesions) which in combination caused a common pathophysiologic abnormality, portal hypertension.
